# Protein degradation and protein synthesis in long-term memory formation

**DOI:** 10.3389/fnmol.2014.00061

**Published:** 2014-06-26

**Authors:** Timothy J. Jarome, Fred J. Helmstetter

**Affiliations:** ^1^Department of Neurobiology, University of Alabama at BirminghamBirmingham, AL, USA; ^2^Department of Psychology, University of Wisconsin-MilwaukeeMilwaukee, WI, USA

**Keywords:** ubiquitin, proteasome, fear conditioning, protein degradation, protein synthesis, amygdala, hippocampus

## Abstract

Long-term memory (LTM) formation requires transient changes in the activity of intracellular signaling cascades that are thought to regulate new gene transcription and *de novo* protein synthesis in the brain. Consistent with this, protein synthesis inhibitors impair LTM for a variety of behavioral tasks when infused into the brain around the time of training or following memory retrieval, suggesting that protein synthesis is a critical step in LTM storage in the brain. However, evidence suggests that protein degradation mediated by the ubiquitin-proteasome system (UPS) may also be a critical regulator of LTM formation and stability following retrieval. This requirement for increased protein degradation has been shown in the same brain regions in which protein synthesis is required for LTM storage. Additionally, increases in the phosphorylation of proteins involved in translational control parallel increases in protein polyubiquitination and the increased demand for protein degradation is regulated by intracellular signaling molecules thought to regulate protein synthesis during LTM formation. In some cases inhibiting proteasome activity can rescue memory impairments that result from pharmacological blockade of protein synthesis, suggesting that protein degradation may control the requirement for protein synthesis during the memory storage process. Results such as these suggest that protein degradation and synthesis are both critical for LTM formation and may interact to properly “consolidate” and store memories in the brain. Here, we review the evidence implicating protein synthesis and degradation in LTM storage and highlight the areas of overlap between these two opposing processes. We also discuss evidence suggesting these two processes may interact to properly form and store memories. LTM storage likely requires a coordinated regulation between protein degradation and synthesis at multiple sites in the mammalian brain.

## Introduction

The ubiquitin-proteasome system (UPS) is a complex network of different ubiquitin ligases and interconnected protein structures involved in the regulation of protein degradation in neurons. This system has been reviewed extensively by others (Hegde, 2010; Mabb and Ehlers, 2010; Bingol and Sheng, 2011), but in general proteins become targeted for degradation through a series of steps in which the small protein modifier ubiquitin is covalently bound to a target substrate. The substrate can acquire anywhere from 1 to 7 ubiquitin modifiers, which link together at specific lysine residues forming polyubiquitin chains. In general, longer ubiquitin chains and lysine-48 linkage provide the maximal signal for degradation (Fioravante and Byrne, 2011) while lysine-63 and M1 linkage (linear ubiquitination) often target substrates for other non-proteolytic functions (Rieser et al., 2013). The ubiquitination process determines what proteins will be targeted for degradation by the proteasome, but the proteasome ultimately controls which of these polyubiquitinated substrates will be degraded.

The catalytic structure in the UPS is the 26S proteasome, which consists of a core (20S) and two regulatory particles (19S) (Bedford et al., 2010). The 20S proteasome is the catalytic core of the proteasome structure and it possesses three types of proteolytic activity controlled by the β-subunits. The activity of the 20S core is regulated by the 19S regulatory particles, which contain the only six ATP-sensitive subunits of the proteasome known as the Rpt subunits. The Rpt6 subunit has received the most attention as it has been shown that increases in its phosphorylation regulates increases in proteasome activity *in vitro* (Bingol et al., 2010; Djakovic et al., 2012) and correlates with increased proteasome activity *in vivo* (Jarome et al., 2013), suggesting that phosphorylation of Rpt6 (at Serine-120) may be the primary regulator of activity-dependent changes in proteasome activity in the brain. Additionally, the 19S proteasome contains numerous deubiquitinating enzymes which generally facilitate the degradation process by removing ubiquitin moieties as the substrate enters the proteasome, thus maintaining the ubiquitin pool (Kowalski and Juo, 2012). However, some deubiquitinating enzymes, such as the ubiquitin-specific protease 14 (USP14), actually seem to inhibit the degradation of certain substrates (Lee et al., 2010; Jin et al., 2012). This suggests that not only does the proteasome degrade polyubiquitinated substrates, but it can actually determine which of these substrates will ultimately be degraded.

In recent years numerous studies have suggested a role for the proteolytic activity of the UPS in activity-dependent synaptic plasticity. For example, bidirectional activity-dependent homeostatic scaling requires UPS-mediated protein degradation (Ehlers, 2003). Interestingly, this proteasome-dependent homeostatic scaling is largely regulated by phosphorylation of the Rpt6 subunit at Serine-120 (Rpt6-S120) (Djakovic et al., 2012) which enhances proteasome activity (Djakovic et al., 2009), suggesting that Rpt6-mediated increases in proteasome activity are critical for activity-dependent synaptic plasticity. Consistent with this, protein degradation is involved in new dendritic spine growth that is regulated by phosphorylation of Rpt6-S120 (Hamilton et al., 2012; Hamilton and Zito, 2013). Additionally, proteasome inhibitors alter long-term potentiation (LTP) in the hippocampus (Fonseca et al., 2006; Dong et al., 2008) and long-term facilitation (LTF) in *Aplysia* (Chain et al., 1999; Lee et al., 2012), suggesting that protein degradation is critical for various forms of synaptic plasticity.

Recently, attention has turned to the potential role of protein degradation in learning-dependent synaptic plasticity. Indeed, there is now convincing evidence that UPS-mediated protein degradation is likely involved in various different stages of memory storage. However, while some studies have suggested potential roles for protein degradation in long-term memory (LTM) formation and storage (Kaang and Choi, 2012), one intriguing question is whether protein degradation is linked to the well-known transcriptional and translational alterations thought to be critical for memory storage in the brain (Johansen et al., 2011). Here, we discuss evidence demonstrating a role for protein degradation and synthesis in the long-term storage of memories in the mammalian brain, highlighting instances in which a requirement for protein degradation correlates with a requirement for protein synthesis. Additionally, we discuss evidence suggesting that both protein degradation and synthesis may be regulated by CaMKII signaling during LTM formation. Collectively, we propose that LTM storage requires coordinated changes in protein degradation and synthesis in the brain, which may be primarily controlled through a CaMKII-dependent mechanism.

## Memory paradigms

A variety of different rodent behavioral paradigms have been used to study the molecular neurobiology of LTM formation, and these behavioral tasks often result in alterations in synaptic plasticity in many different brain regions. This review will focus mostly on fear-related conditioning paradigms, but will also discuss results from the Morris water maze (MWM) and the object recognition paradigm. One of the most common rodent behavioral procedures is Pavlovian fear conditioning, in which a neutral conditional stimulus (CS) becomes associated with a noxious or aversive unconditional stimulus (UCS). As a result of this association, the CS can elicit an emotional response based on the memory of the UCS. A simple form of Pavlovian fear conditioning is contextual fear conditioning, in which rodents learn to fear the specific context or training environment in which the UCS occurred. Memories in this paradigm require an intact and active amygdala and hippocampus for their formation and long-term storage (Kim and Fanselow, 1992; Phillips and Ledoux, 1992). Auditory delay fear conditioning involves a discrete auditory cue (CS) that coterminates with the UCS. Memories formed in this paradigm require an intact and active amygdala, but unlike contextual fear conditioning, do not require the hippocampus (Helmstetter, 1992a,b; Phillips and Ledoux, 1992; Ledoux, 2000). A more complex form of fear conditioning is auditory trace fear conditioning in which the auditory CS predicts the UCS but the two stimuli are separated in time. The introduction of this “trace interval” recruits the prefrontal cortex (Gilmartin and McEchron, 2005b; Gilmartin et al., 2013b). Trace fear conditioning also requires the hippocampus and amygdala (Gilmartin and McEchron, 2005a; Kwapis et al., 2011; Gilmartin et al., 2012). Fear conditioning can be measured in several ways including behavioral observation of freezing (e.g., Helmstetter, 1992a,b) or CS modulation of reflex responses (Hitchcock and Davis, 1991; Rosen et al., 1991). Inhibitory avoidance is another popular aversive learning procedure. In this task, rodents are placed into the lit (white) compartment of a black-white shuttle box. Once the partition is opened, the animal will go to the dark (black) side of the box where it will receive a shock. Animals will then learn to avoid the dark compartment to prevent receiving the shock again. Memories formed using this paradigm require the amygdala and hippocampus for their long-term storage (Taubenfeld et al., 2001; Milekic et al., 2007). Finally, another form of aversive classical conditioning is conditioned taste aversion, in which rodents will acquire an aversion to a specific food due to experiencing illness associated with it. Memories formed using this paradigm require the amygdala and insular cortex for their long-term storage (Rodriguez-Ortiz et al., 2011). In general, memories formed through aversive conditioning require the amygdala but the contribution of the hippocampus, prefrontal cortex and insular cortex depend on the specific behavioral paradigm used.

Two of the most common non-shock based spatial paradigms used to study LTM formation are the MWM and object recognition procedures. In the MWM, a spatial paradigm that can also be stressful and aversive (D'Hooge and De Deyn, 2001), rodents are typically placed into a pool where a hidden platform is positioned in one of four quadrants, each of which has specific spatial cues surrounding it. The animal is given several trials to learn where the platform is, and its latency to swim to the platform will decrease as it learns the task. On the probe day, the platform is removed and the amount of time the animal spends searching each quadrant is measured. If the animal learned the task, it will spend a majority of its time searching the target quadrant where the platform had been during training. Due to the spatial nature of the task, memories acquired using this paradigm require the hippocampus (Artinian et al., 2008). In the objection recognition paradigm, a non-aversive form of spatial learning, rodents are allowed to explore two objects for a period of time. At later test one of the objects is replaced with a new object and the amount of time the animal spends exploring the objects is recorded Object memory is indicated by more time spent exploring the novel object during the test phase. As in the MWM, memories formed in this paradigm require the hippocampus for their formation and long-term storage (Rossato et al., 2007).

## The role of protein synthesis in memory storage

For several decades there has been a general consensus that *de novo* protein synthesis is critical for the formation and stability of LTM (Davis and Squire, 1984; Helmstetter et al., 2008; Johansen et al., 2011). Consistent with this, numerous studies using pharmacological, molecular, and genetic approaches have implicated a role for increased transcriptional and translational regulation in various brain regions during LTM formation for a variety of different behavioral tasks. Table 1 summarizes some the findings for inhibitors of gene transcription and protein synthesis on LTM formation and stability following retrieval. Some of the first evidence suggesting that new protein synthesis may be necessary for LTM after fear conditioning came from a study showing that infusions of a broad spectrum mRNA synthesis inhibitor in the amygdala impaired memory for auditory and contextual fear conditioning (Bailey et al., 1999). Consistent with this, infusions of broad spectrum inhibitors of gene transcription or protein synthesis into the amygdala can impair LTM for auditory delay fear conditioning, auditory trace fear conditioning, contextual fear conditioning, fear potentiated startle, inhibitory avoidance, and conditioned taste aversion memories (Schafe and Ledoux, 2000; Bahar et al., 2003; Lin et al., 2003; Yeh et al., 2006; Milekic et al., 2007; Jarome et al., 2011; Kwapis et al., 2011), suggesting that increased protein synthesis is critical for the storage of fear memories in the amygdala (Hoeffer et al., 2011). Inhibiting protein synthesis in the hippocampus impairs LTM for contextual fear conditioning, MWM spatial memories, inhibitory avoidance memories, and object recognition memories (Bourtchouladze et al., 1998; Taubenfeld et al., 2001; Rossato et al., 2007; Artinian et al., 2008), while inhibiting protein synthesis impairs LTM for conditioned taste aversion memories in the insular cortex and trace fear memories in the medial prefrontal cortex (Blum et al., 2006; Moguel-Gonzalez et al., 2008; Reis et al., 2013). Additionally, infusions of more selective inhibitors of protein synthesis which block mTOR-mediated translation impair fear memory and object recognition memory formation in the amygdala and hippocampus, supporting that *de novo* translation is critical for LTM formation in these regions (Parsons et al., 2006b; Gafford et al., 2011; Jobim et al., 2012a,b). Collectively, these results suggest new protein synthesis is required for the processes of memory consolidation.

**Table 1:** **The role of protein synthesis in memory consolidation and reconsolidation**.

**Structure**	**Task**	**Manipulation**	**Consolidation or reconsolidation**	**Effect on memory**	**References**
Amygdala	Auditory/contextual fear conditioning	Anisomycin	Consolidation	Impaired	Schafe and Ledoux, 2000
Amygdala	Auditory/contextual fear conditioning	Rapamycin	Consolidation	Impaired	Parsons et al., 2006b
Amygdala	Trace fear conditioning	Anisomycin	Consolidation	Impaired	Kwapis et al., 2012
Amygdala	Conditioned taste aversion	Anisomycin	Consolidation	Impaired	Bahar et al., 2003
Amygdala	Fear potentiated startle	Anisomycin	Consolidation	Impaired	Yeh et al., 2006
Amygdala	Inhibitory avoidance	Anisomycin	Consolidation	Impaired	Milekic et al., 2007
Hippocampus	Contextual fear conditioning	Anisomycin	Consolidation	Impaired	Bourtchouladze et al., 1998
Hippocampus	Contextual fear conditioning	Rapamycin	Consolidation	Impaired	Gafford et al., 2011
Hippocampus	Inhibitory avoidance	Anisomycin	Consolidation	Impaired	Taubenfeld et al., 2001
Hippocampus	Morris water Maze	Anisomycin	Consolidation	Impaired	Artinian et al., 2008
Hippocampus	Object recognition memory	Anisomycin	Consolidation	Impaired	Rossato et al., 2007
Hippocampus	Object recognition memory	Rapamycin	Consolidation	Impaired	Jobim et al., 2012b
Prefrontal cortex	Trace fear conditioning	Anisomycin	Consolidation	Impaired	Reis et al., 2013
Insular cortex	Conditioned taste aversion	Anisomycin	Consolidation	Impaired	Moguel-Gonzalez et al., 2008
Amygdala	Auditory fear conditioning	Anisomycin	Reconsolidation	Impaired	Nader et al., 2000
Amygdala	Auditory fear conditioning	Rapamycin	Reconsolidation	Impaired	Parsons et al., 2006b
Amygdala	Contextual fear conditioning	Anisomycin	Reconsolidation	Impaired	Jarome et al., 2011
Amygdala	Trace fear conditioning	?	Reconsolidation	?	?
Amygdala	Conditioned taste aversion	Anisomycin	Reconsolidation	Impaired	Rodriguez-Ortiz et al., 2012
Amygdala	Fear potentiated startle	?	Reconsolidation	?	?
Amygdala	Inhibitory avoidance	Anisomycin	Reconsolidation	Impaired	Milekic et al., 2007
Hippocampus	Contextual fear conditioning	Anisomycin	Reconsolidation	Impaired	Debiec et al., 2002
Hippocampus	Contextual fear conditioning	Rapamycin	Reconsolidation	Impaired	Gafford et al., 2011
Hippocampus	Inhibitory avoidance	Anisomycin	Reconsolidation	No effect	Taubenfeld et al., 2001
Hippocampus	Morris water Maze	Anisomycin	Reconsolidation	Impaired	Artinian et al., 2008
Hippocampus	Object recognition memory	Anisomycin	Reconsolidation	Impaired	Rossato et al., 2007
Prefrontal cortex	Trace fear conditioning	?	Reconsolidation	?	?
Insular cortex	Conditioned taste aversion	Anisomycin	Reconsolidation	No effect	Garcia-DeLaTorre et al., 2009

?,Denotes that the role of protein synthesis has not been tested for this type of memory during the indicated stage of memory storage.

In addition to a role for new protein synthesis in LTM consolidation, numerous studies have shown that the use or retrieval of an established memory results in a second phase of increased protein synthesis, a process referred to as memory reconsolidation. Protein synthesis inhibitors infused into the amygdala can impair the reconsolidation of auditory delay fear memories, contextual fear memories, inhibitory avoidance memories, and conditioned taste aversion memories (Nader et al., 2000; Parsons et al., 2006a; Milekic et al., 2007; Jarome et al., 2011, 2012; Rodriguez-Ortiz et al., 2012). Additionally, inhibiting protein synthesis in the hippocampus impairs the reconsolidation of contextual fear memories (Debiec et al., 2002; Lee et al., 2008; Gafford et al., 2011), MWM spatial memories (Artinian et al., 2008), and object recognition memories (Rossato et al., 2007), though it has no effect on inhibitory avoidance memories in the hippocampus (Taubenfeld et al., 2001) or conditioned taste aversion memories in the insular cortex (Garcia-DeLaTorre et al., 2009). These results indicate that new protein synthesis is a necessary step in the transfer of a retrieved memory back to long-term storage, suggesting that both the consolidation and reconsolidation of memories requires *de novo* protein translation in several brain regions.

Consistent with the evidence from the broad spectrum inhibitors, several studies have implicated a role for specific intracellular signaling molecules thought to be “upstream” of protein synthesis in LTM formation and storage (Johansen et al., 2011). For example, inhibition of NMDA receptor (NMDAR) function impairs the consolidation of auditory delay fear and contextual fear memories, fear potentiated startle, and conditioned taste aversion memories in the amygdala (Walker and Davis, 2000; Yasoshima et al., 2000; Rodrigues et al., 2001), auditory trace and contextual fear memories in the prefrontal cortex (Gilmartin and Helmstetter, 2010; Gilmartin et al., 2013a), contextual fear memories, MWM and objection recognition spatial memories in the hippocampus (Liang et al., 1994; Izquierdo et al., 1999; Czerniawski et al., 2012; Da Silva et al., 2013; Warburton et al., 2013), and conditioned taste aversion memories in the insular cortex (Escobar et al., 1998). Inhibition of signaling molecules thought to be downstream of NMDAR activity but upstream of protein synthesis such as Protein Kinase A, ERK-MAPK, and CaMKII impairs memory consolidation for auditory fear memories, contextual fear memories, inhibitory avoidance memories, MWM spatial memories, and conditioned taste aversion memories (Schafe and Ledoux, 2000; Schafe et al., 2000; Sacchetti et al., 2001; Koh et al., 2002; Quevedo et al., 2004; Rodrigues et al., 2004; Leon et al., 2010; Ota et al., 2010; Chen et al., 2012; Halt et al., 2012). Many of these signaling molecules are thought to regulate the transient changes in gene expression hypothesized to be critical for LTM formation in the brain. Consistent with this, epigenetic modifications such as acetylation, phosphorylation, and methylation of histones and DNA methylation are critical for LTM formation in the amygdala and hippocampus (Levenson et al., 2004; Lubin et al., 2008; Gupta et al., 2010; Maddox and Schafe, 2011; Jarome and Lubin, 2013), suggesting that LTM formation requires dynamic changes in gene transcription and protein translation in multiple regions of the brain.

## The role of protein degradation in memory storage

The idea that protein degradation could contribute to activity-dependent synaptic plasticity was first identified more than a decade ago by a study demonstrating that the induction of LTF in *Aplysia* resulted in increased expression of *Ap-uch*, which encodes a ubiquitin C-terminal hydrolase, and that a loss of this gene impaired LTF (Hegde et al., 1997). Additionally, application of the proteasome inhibitor lactacystin impairs LTF (Chain et al., 1999), suggesting that functional proteasome activity is critical for synaptic plasticity. Consistent with this identified role of ubiquitin-proteasome mediated protein degradation in activity-dependent synaptic plasticity, homeostatic changes in synaptic strength that result from chronic stimulation or inhibition of cultured hippocampal neurons requires activity of the UPS (Ehlers, 2003). This activity-dependent homeostatic scaling requires phosphorylation of the proteasome subunit Rpt6 at Serine-120, a CaMKII target site (Djakovic et al., 2009; Bingol et al., 2010). Remarkably, enhancements in Rpt6 phosphorylation is sufficient to drive long-term changes in synaptic strength (Djakovic et al., 2012) and new dendritic spine growth *in vitro* (Hamilton et al., 2012), suggesting that protein degradation is a critical regulator of synaptic plasticity. Furthermore, numerous studies have indicated a role for protein degradation in LTP, the proposed cellular analog of memory (for review, see Hegde, 2010). For example, inhibiting protein degradation or protein synthesis individually impairs late-LTP (Fonseca et al., 2006), suggesting that protein degradation is critical for maintaining LTP following its induction. Interestingly, proteasome inhibitors can also enhance the induction of LTP (Dong et al., 2008), an effect that is largely due to the proteasomes targeting of translational activators during LTP induction (Dong et al., 2014). Thus, it is well-established that protein degradation is a critical regulator of synaptic plasticity *in vitro*.

While not considered in traditional memory models, the theory that protein degradation accompanies changes in protein synthesis during LTM formation has become increasingly popular. Indeed, numerous studies now clearly demonstrate a role for UPS-mediated protein degradation in LTM formation and storage in neurons (Felsenberg et al., 2012; Kaang and Choi, 2012; Jarome and Helmstetter, 2013). Interestingly, strong evidence suggests that changes in protein degradation are correlated with changes in protein synthesis in specific brain regions during both memory consolidation and reconsolidation. Table 2 summarizes the findings for genetic and pharmacological manipulations of ubiquitin-proteasome activity on LTM formation and stability following retrieval. Proteasome inhibitors infused in the amygdala alter LTM for contextual and auditory fear conditioning (Jarome et al., 2011), fear potentiated startle (Yeh et al., 2006) but not conditioned taste aversion memories (Rodriguez-Ortiz et al., 2011). Additionally, proteasome inhibitors impair MWM and inhibitory avoidance memories in the hippocampus (Lopez-Salon et al., 2001; Artinian et al., 2008), trace fear memories in the prefrontal cortex (Reis et al., 2013), and conditioned taste aversion memories in the insular cortex (Rodriguez-Ortiz et al., 2011). These results suggest a strong overlap between the protein degradation and synthesis processes during LTM formation. Consistent with this, fear conditioning simultaneously increases protein polyubiquitination and mTOR phosphorylation in the amygdala 1 h after behavioral training (Jarome et al., 2011), a time when protein synthesis is increased in the amygdala (Hoeffer et al., 2011), suggesting a potential overlap between protein degradation and synthesis processes. However, inhibiting proteasome activity in the hippocampus does not alter LTM for contextual fear conditioning. Consistent with this finding, a genetic loss of a ubiquitin E3 ligase alters memory for amygdala but not hippocampus dependent fear memories (Pick et al., 2013a,b), suggesting that there is not a perfect overlap between the protein degradation and synthesis processes during LTM formation. In general, these results do suggest though that protein degradation and protein synthesis are both critical for LTM formation in the same brain regions and they likely overlap in time following behavioral training.

**Table 2:** **The role of protein degradation in memory consolidation and reconsolidation**.

**Structure**	**Task**	**Manipulation**	**Consolidation/reconsolidation**	**Effect on memory**	**References**
Amygdala	Auditory/contextual fear conditioning	Clasto-lactacystin beta-lactone	Consolidation	Impaired	Jarome et al., 2011
Amygdala	Auditory/contextual fear conditioning	Genetic deletion of E3 ligase	Consolidation	Impaired	Pick et al., 2013a
Amygdala	Trace fear conditioning	?	Consolidation	?	?
Amygdala	Conditioned taste aversion	Lactacystin	Consolidation	Impaired	Rodriguez-Ortiz et al., 2011
Amygdala	Fear potentiated startle	Lactacystin	Consolidation	Impaired	Yeh et al., 2006
Amygdala	Inhibitory avoidance	?	Consolidation	?	?
Hippocampus	Contextual fear conditioning	Clasto-lactacystin beta-lactone	Consolidation	No effect	Lee et al., 2008
Hippocampus	Contextual fear conditioning	Genetic deletion of E3 ligase	Consolidation	No effect	Pick et al., 2013a
Hippocampus	Inhibitory avoidance	Lactacystin	Consolidation	Impaired	Lopez-Salon et al., 2001
Hippocampus	Morris water Maze	Lactacystin	Consolidation	Impaired	Artinian et al., 2008
Hippocampus	Object recognition memory	?	Consolidation	?	?
Prefrontal cortex	Trace fear conditioning	Clasto-lactacystin beta-lactone	Consolidation	Impaired	Reis et al., 2013
Insular cortex	Conditioned taste aversion	Lactacystin	Consolidation	Impaired	Rodriguez-Ortiz et al., 2011
Amygdala	Auditory fear conditioning	Clasto-lactacystin beta-lactone	Reconsolidation	Prevented effects of protein synthesis inhibitors	Jarome et al., 2011
Amygdala	Contextual fear conditioning	Clasto-lactacystin beta-lactone	Reconsolidation	Prevented effects of protein synthesis inhibitors	Jarome et al., 2011
Amygdala	Trace fear conditioning	?	Reconsolidation	?	?
Amygdala	Conditioned taste aversion	Lactacystin	Reconsolidation	No effect	Rodriguez-Ortiz et al., 2011
Amygdala	Fear potentiated startle	?	Reconsolidation	?	?
Amygdala	Inhibitory avoidance	?	Reconsolidation	?	?
Hippocampus	Contextual fear conditioning	Clasto-lactacystin beta-lactone	Reconsolidation	Prevented effects of protein synthesis inhibitors	Lee et al., 2008
Hippocampus	Inhibitory avoidance	?	Reconsolidation	?	?
Hippocampus	Morris water Maze	Lactacystin	Reconsolidation	Impaired	Artinian et al., 2008
Hippocampus	Object recognition memory	?	Reconsolidation	?	?
Prefrontal cortex	Trace fear conditioning	?	Reconsolidation	?	?
Insular cortex	Conditioned taste aversion	Lactacystin	Reconsolidation	Impaired	Rodriguez-Ortiz et al., 2011

?,Denotes that the role of protein degradation has not been tested for this type of memory during the indicated stage of memory storage.

Protein degradation is also critical for the reconsolidation of fear memories, though much less is known about the role of the UPS in this stage of memory storage. For example, proteasome inhibitors alter the reconsolidation of auditory and contextual fear memories in the amygdala (Jarome et al., 2011), contextual fear memories and MWM memories in the hippocampus (Artinian et al., 2008; Lee, 2008, 2010; Lee et al., 2008) and conditioned taste aversion memories in the insular cortex (Rodriguez-Ortiz et al., 2011), though it is currently unknown if protein degradation regulates the reconsolidation of trace fear memories, inhibitory avoidance memories, and object recognition memories in the brain. Despite this, there does appear to be a clear overlap between the protein degradation and protein synthesis processes during memory reconsolidation as manipulation of protein degradation in the amygdala and hippocampus prevent the effectiveness of protein synthesis inhibitors at disrupting LTM for fear conditioning tasks. Collectively, these results suggest a strong correlation between protein degradation and protein synthesis during the reconsolidation process.

In addition to the protein degradation function, non-proteolytic functions of the UPS are also critical for LTM and correlate with the demand for increased protein synthesis. For example, non-proteolytic monoubiquitination of the cytoplasmic polyadenylation element binding protein 3 (CPEB3) by the E3 ligase Neuralized1 is critical for the consolidation of hippocampus dependent memories (Pavlopoulos et al., 2011). Interestingly, monoubiquitination of CPEB3 by Neuralized1 was critical for activity dependent increases in GluR1 and GluR2, suggesting that non-proteolytic ubiquitination regulates protein synthesis during LTM formation. Additionally, recent evidence suggests that the USP14 is a critical regulator of LTM formation in the amygdala (Jarome et al., 2014). USP14 acts as a negative regulator of protein turnover (Lee et al., 2010) and regulates presynaptic plasticity *in vitro* (Wilson et al., 2002; Walters et al., 2008; Bhattacharyya et al., 2012), suggesting that USP14 regulates memory formation through a mechanism independent of protein degradation. These studies highlight that both proteolytic and non-proteolytic functions of the UPS regulate LTM formation in the amygdala and hippocampus, suggesting that overall changes in ubiquitin-proteasome activity correlates with the increased demand for *de novo* protein synthesis during memory consolidation.

## The linking of protein degradation and synthesis through NMDA-CaMKII signaling

In addition to the potential overlap between protein degradation and synthesis in the brain during LTM formation, both of these mechanisms are likely regulated by a similar signaling pathway: NMDA receptor (NMDAR) dependent changes in CaMKII activity. As stated above, similar to protein degradation and synthesis, NMDAR activity at the time of training has been shown to be critical for the formation of different types of memories in various brain regions. NMDAR activity is thought to regulate the changes in protein synthesis necessary for LTM formation by altering the activity of a number of downstream signaling pathways (Johansen et al., 2011; Jarome and Helmstetter, 2013). One such signaling molecule is CaMKII which is thought to be involved in changes in gene transcription and protein synthesis through its regulation of CREB (Wayman et al., 2008), a critical regulator of LTM formation and stability in the neurons (Josselyn et al., 2001; Han et al., 2007, 2008, 2009). Indeed, numerous studies have suggested that CaMKII is critical for LTM formation and that this requirement for CaMKII signaling overlaps with the requirement for protein degradation and synthesis during memory consolidation (Barros et al., 1999; Rodrigues et al., 2004; Von Hertzen and Giese, 2005; Halt et al., 2012; Da Silva et al., 2013). Collectively, these results suggest that changes in NMDAR and CaMKII activity correlate with changes in both protein degradation and protein synthesis during LTM formation in neurons.

In addition to overlapping with an increased need for protein degradation, recent evidence suggests that NMDAR and CAMKII activity can regulate UPS-mediated protein degradation during LTM in neurons (Jarome et al., 2011, 2013). Inhibiting the activity of NR2B-containing NMDARs, a manipulation which impairs LTM (Rodrigues et al., 2001), prevents learning-dependent increases in degradation-specific polyubiquitination in the amygdala (Jarome et al., 2011). This supports the previously identified *in vitro* relationship between NMDAR and ubiquitin-proteasome activity (Bingol and Schuman, 2006; Bingol et al., 2010) and suggests that NMDAR activity regulates changes in protein degradation in neurons during memory consolidation. Additionally, inhibiting CaMKII signaling in the amygdala during LTM formation prevents learning-induced increases in proteasome activity (Jarome et al., 2013), suggesting that CaMKII signaling is critical for changes in protein degradation in neurons during memory consolidation. Interestingly, pharmacological manipulation of CaMKII also prevented learning-induced increases in the phosphorylation of the proteasome regulatory subunit Rpt6, which is critical for changes in proteasome activity, synaptic plasticity and dendritic spine growth *in vitro* (Djakovic et al., 2009, 2012; Hamilton et al., 2012), suggesting that CaMKII may regulate protein degradation through its actions on the proteasome ATPase subunits. Importantly, manipulation of protein kinase A (PKA), another NMDAR-dependent signaling molecule that is critical for LTM formation in neurons (Schafe and Ledoux, 2000; Tronson et al., 2006), did not alter the changes in proteasome phosphorylation and activity during memory consolidation. This demonstrates that PKA, which can regulate protein degradation *in vitro* (Upadhya et al., 2006; Zhang et al., 2007), does not regulate protein degradation during memory formation and suggests that not all NMDAR-dependent signaling pathways regulate ubiquitin-proteasome activity during LTM formation. Collectively, these results suggest that NMDA-CaMKII-dependent changes in ubiquitin-proteasome mediated protein degradation are critical for LTM formation in neurons, suggesting that protein degradation and synthesis could be linked during memory formation by CaMKII signaling, however, it is still unknown if CaMKII actually does regulate protein synthesis during memory formation.

## What comes first, degradation or synthesis?

A majority of the studies discussed here reveal a strong correlation between protein degradation and synthesis during LTM formation. This leads to one important question: Which comes first? While the exact relationship between protein degradation and synthesis during memory formation currently remains equivocal, the available evidence suggests that protein degradation likely regulates protein synthesis. For example, fear conditioning leads to an increase in polyubiquitinated proteins being targeted for degradation by the proteasome (Jarome et al., 2011). While a majority of the proteins being targeted by the proteasome for degradation remain unknown, the RNAi-induced Silencing Complex (RISC) factor MOV10 has been identified as a target of the proteasome during increases in activity-dependent protein degradation *in vitro* (Banerjee et al., 2009) and following behavioral training and retrieval *in vivo* (Jarome et al., 2011). Increases in the degradation of MOV10 are associated with increased protein synthesis *in vitro*, suggesting that the proteasome could regulate protein synthesis during LTM formation through the removal of translational repressor proteins such as various RISC factors. However, it is currently unknown if the selective degradation of MOV10, or any RISC factor, is critical for memory formation in neurons. Nonetheless, studies such as these provide indirect evidence that protein degradation by the UPS could regulate protein synthesis during memory formation in the brain.

Some of the best evidence that protein degradation may be upstream of protein synthesis during memory storage comes from studies examining memory reconsolidation following retrieval. For example, inhibiting proteasome activity can prevent the memory impairments that normally result from post-retrieval blockade of protein synthesis in the hippocampus, amygdala, and nucleus accumbens (Lee et al., 2008; Jarome et al., 2011; Ren et al., 2013) as well as during LTF in aplysia (Lee et al., 2012), suggesting that protein degradation is upstream of protein synthesis during memory reconsolidation. This remains some of the best evidence directly linking protein degradation to protein synthesis during memory storage, but it is possible that the rescue of memory impairments in the face of protein synthesis inhibition may occur as an indirect consequence of blocking protein degradation rather than a direct effector. Additionally, some studies find that proteasome inhibitors impair memory reconsolidation when administered on their own (Artinian et al., 2008; Rodriguez-Ortiz et al., 2011), suggesting that this relationship between protein degradation and synthesis may not exist for all types of memories. However, in cell cultures protein degradation has been shown to regulate mTOR activity (Ghosh et al., 2008), a translational control pathway critical for memory formation and storage (Parsons et al., 2006b; Gafford et al., 2011), though it is unknown if this relationship exists *in vivo* during memory formation. Collectively, the current evidence suggests that protein degradation may determine the requirement for protein synthesis during memory storage, though this relationship has never been directly proven.

While it is currently not known if protein degradation regulates protein synthesis during memory formation, one recent study found that protein degradation regulates protein synthesis during LTP, a proposed cellular analog of memory (Dong et al., 2014). Inhibiting proteasome activity enhances the induction but impairs the maintenance of LTP (Dong et al., 2008). Surprisingly, the enhanced induction of LTP following proteasome inhibition can be blocked by inhibiting the activity of several proteins involved in mTOR-mediated protein synthesis, suggesting that the proteasome targets translational activators during LTP induction. However, proteasome inhibitors cause an increase in translational repressors during L-LTP, suggesting that the proteasome acts to enhance protein synthesis during LTP maintenance. Collectively, these results demonstrate for the first time that protein degradation can directly regulate protein synthesis during activity-dependent synaptic plasticity. While it is currently unknown if the proteasome targets similar proteins during memory formation, this study provides some of the best evidence to date that protein degradation may regulate protein synthesis during learning-dependent synaptic plasticity.

## Regulation of memory storage by protein degradation and synthesis

While the functional significance of the observed overlap between protein degradation and synthesis during LTM formation is unknown, it is possible that a coordinated balance between protein degradation and synthesis may be necessary for memory formation and storage in neurons (Jarome and Helmstetter, 2013). This theory has been described extensively elsewhere, but in this model CaMKII-dependent increases in proteasome activity leads to reductions in a number of different proteins that normally repress transcription and translation or prevent alterations to the postsynaptic structure. Consistent with this, the proteasome targets the RISC factor MOV10 and synaptic scaffold Shank following behavioral training and memory retrieval (Lee et al., 2008; Jarome et al., 2011), and inhibiting proteasome activity can prevent learning-induced changes in GluR2 expression at synapses (Ren et al., 2013). Thus, the changes in protein degradation could be necessary to remove repressor proteins that prevent the dynamic changes to the postsynaptic structure that characterize memory storage in neurons (Ostroff et al., 2010) while protein synthesis could produce the proteins that are necessary for these changes. In addition, an attractive new addition to this model is that protein degradation and synthesis could regulate dynamic changes in the atypical protein kinase C isoform PKMζ during memory consolidation. A majority of studies have shown that inhibiting PKMζ impairs LTM even after the consolidation process has completed (Pastalkova et al., 2006; Shema et al., 2007; Kwapis et al., 2009, 2012), though a few exceptions have been noted (Parsons and Davis, 2011; Volk et al., 2013), suggesting that PKMζ regulates the maintenance of memories in neurons (Sacktor, 2012; Kwapis and Helmstetter, 2013). Interestingly, one recent study (Vogt-Eisele et al., 2014) found that protein degradation can regulate PKMζ levels during memory storage through targeting of KIBRA (Kidney/BRAin protein). Since behavioral training is thought to increase PKMζ levels in the brain (Sacktor, 2012), this suggests that a balance between protein degradation and synthesis may control LTM formation and storage through regulation of PKMζ levels. Whether a balance between protein degradation and synthesis is necessary for learning-dependent changes in PKMζ levels will be of interest in future studies.

## Protein degradation and synthesis as independent processes during memory storage

While the evidence discussed here suggests that protein degradation and protein synthesis may be interconnected processes necessary for memory formation and storage, an alternative theory is that these are actually opposing processes and that memory formation requires an appropriate balance between them. For example, both proteasome and protein synthesis inhibitors impair LTP when applied individually, but actually rescue these deficits when applied simultaneously (Fonseca et al., 2006). This would suggest that memory impairments that result from blocking protein degradation or synthesis individually are caused by an inappropriate balance between the two processes. While an intriguing theory, few studies have directly tested this *in vivo* during memory formation as most times proteasome inhibitors are given alone and not in combination with protein synthesis inhibitors. Despite this, some studies suggest that an altered balance between protein degradation and synthesis cannot account for memory impairments following disruption of the UPS. For example, proteasome inhibitors infused into the amygdala result in similar memory impairments for a fear conditioning task as those produced by the broad spectrum protein synthesis inhibitor anisomycin and simultaneous infusion of both inhibitors does not rescue these impairments (Jarome et al., 2011). This suggests that an inappropriate balance between protein degradation and synthesis likely cannot account for memory deficits observed following infusions of either inhibitor individually. However, simultaneous blockade of protein degradation and synthesis can rescue memory impairments that results from blocking protein synthesis individually following memory retrieval (Lee et al., 2008; Jarome et al., 2011), though in these cases proteasome inhibitors have no effect on memory on their own. This would lend more toward the theory that protein degradation is upstream of protein synthesis during memory storage following retrieval. Thus, while the idea that protein degradation and protein synthesis are independent processes and a balance between them is the primary factor that underlies memory formation is intriguing, very few studies have directly tested this to date.

## Summary and future directions

The studies reviewed here suggest a clear correlation between protein degradation and protein synthesis in LTM formation in the brain. However, we are currently far from understanding the exact relationship between these two opposing processes during in memory. As discussed above, the current evidence suggests that protein degradation and synthesis are both important regulators of LTM for auditory delay and trace fear conditioning, contextual fear conditioning, inhibitory avoidance, conditioned taste aversion, MWM, and object recognition memories and simultaneously occur in multiple brain regions including the amygdala, hippocampus, prefrontal cortex, and insular cortex. However, it is unknown if this relationship holds for all brain regions and behavioral tasks. For example, contextual and trace fear memories also require the retrosplinal cortex for their long-term storage (Kwapis et al., 2014), though it is unknown if protein degradation and protein synthesis are required. Future research should determine which behavioral tasks and brain regions require protein degradation and protein synthesis to better determine the extent of overlap in these processes. More importantly, future research should focus on determining if and how protein degradation regulates activity driven protein synthesis during memory formation in the already identified instances in which these two processes locally correlate. Such information is critical to determine not only if protein degradation and synthesis are dissociable during memory formation, but also what the functional role of the changes in protein degradation is. Furthermore, identifying the targets of the proteasome during increased protein degradation levels will also help determine the functional role of protein degradation during memory formation. Understanding the exact relationship between protein degradation and synthesis is a critical step in understanding the molecular neurobiology of LTM formation and storage in neurons.

Additionally, future studies should focus on how CaMKII signaling regulates increases in proteasome activity during memory formation and whether this occurs through Rpt6 phosphorylation. Considering that CaMKII is the only intracellular signaling molecule known to regulate protein degradation during LTM formation (Jarome et al., 2013) and is thought to regulate protein synthesis, it is critical to understand how changes in CaMKII activity contribute to the appropriate regulation of protein degradation and synthesis during LTM formation in neurons. Thus, CaMKII might serve as an attractive target to better understand the role of protein degradation in LTM formation and its relationship to *de novo* protein synthesis. Finally, future studies should focus on identifying the non-proteolytic functions of the UPS during memory formation and storage. Currently, only two non-proteolytic roles for ubiquitin-proteasome activity exist (Pavlopoulos et al., 2011), though it is likely that many more remain to be discovered. Considering that one of these non-proteolytic functions has been suggested to regulate some protein synthesis, the degradation-independent functions of the UPS may prove to be among the most important regulators of translational regulation by the UPS.

## Conclusions

Here, we reviewed a number of studies suggesting that changes in protein degradation correlate with changes in protein synthesis during memory formation and storage in neurons. Additionally, we discussed evidence demonstrating that protein degradation is regulated by NMDAR activity and the intracellular signaling molecule CaMKII, two well-known regulators of memory formation that are thought to regulate protein synthesis, suggesting that protein degradation and synthesis may be linked through CaMKII signaling. Finally, we reviewed evidence demonstrating that protein degradation may be upstream of protein synthesis during LTM formation, suggesting that protein degradation may supersede protein synthesis during the memory consolidation process. Collectively, the studies outlined in this review suggest that protein degradation and protein synthesis are not likely two independent regulators of LTM formation but rather directly interact to modify active synapses and store memory for a variety of different behavioral tasks.

### Conflict of interest statement

The authors declare that the research was conducted in the absence of any commercial or financial relationships that could be construed as a potential conflict of interest.
